# Morphological and Whole-Word Semantic Processing Are Distinct: Event Related Potentials Evidence From Spoken Word Recognition in Chinese

**DOI:** 10.3389/fnhum.2019.00133

**Published:** 2019-04-17

**Authors:** Lijuan Zou, Jerome L. Packard, Zhichao Xia, Youyi Liu, Hua Shu

**Affiliations:** ^1^School of Psychology and Education, Zaozhuang University, Zaozhuang, China; ^2^Beckman Institute, University of Illinois, Urbana, IL, United States; ^3^State Key Laboratory of Cognitive Neuroscience and Learning & IDG/McGovern Institute for Brain Research, Beijing Normal University, Beijing, China; ^4^Center for Collaboration and Innovation in Brain and Learning Sciences, Beijing Normal University, Beijing, China

**Keywords:** morphological processing, whole-word semantic processing, ERP, spoken word recognition, Chinese

## Abstract

Behavioral and imaging studies in alphabetic languages have shown that morphological processing is a discrete and independent element of lexical access. However, there is no explicit marker of morphological structure in Chinese complex words, such that the extent to which morpheme meaning is related to word meaning is unknown. Event-related potentials (ERPs) were used in the present study to investigate the dissociation of morphemic and whole-word meaning in an auditory-auditory primed lexical decision task. All the prime and target words are compounds consisting of two Chinese morphemes. The relationship between morpheme and whole-word meaning was manipulated while controlling the phonology and orthography of the first syllable in each prime-target pair. A clear dissociation was found between morphemic and whole-word meaning on N400 amplitude and topography. Specifically, sharing a morpheme produced a larger N400 in the anterior-central electrode sites, while sharing whole-word meaning produced a smaller N400 in central-posterior electrode sites. In addition, the morphological N400 effect was negatively correlated with the participants’ reading ability, with better readers needing less orthographic information to distinguish different morphemes in compound words. These findings indicate that morphological and whole-word meaning are dissociated in spoken Chinese compound word recognition and that even in the spoken language modality, good readers are better able to access the meaning of individual morphemes in Chinese compound word processing.

## Introduction

Spoken word recognition requires the analysis of acoustic-phonetic information into constituent morphemes and words, which involves phonological encoding, orthographic activation and semantic integration. In Chinese, polysemous morphemes are prevalent in compound words. A given character sharing the same phonology and orthography might correspond to several different morphemes. Therefore, it is important to distinguish the different morphemes that are pronounced and written identically during spoken language comprehension. Unlike English and other Western languages in which morphologically complex words contain inflectional and derivational affixes (Crepaldi et al., 2016), there is no morphological marker on the morphemes of Chinese compound words. Morphemic processing in Chinese compound words comes from the interaction between constituent morpheme meaning and whole word semantics. To the best of our knowledge, no study has examined whether the electrophysiological mechanisms underlying Chinese morphological processing is dissociated from whole-word semantic processing in the auditory modality.

Evidence from imaging studies of alphabetic languages has shown that morphological processing is an important and independent component affecting the organization of the mental lexicon. Previous research has shown that morphological processing has a specific neural signature that cannot be reduced to the joint activation of form and meaning (Rastle et al., 2004; Beyersmann et al., 2014). One common approach to test the importance of morphological processing has been to compare the priming produced by morphologically related words with those produced by other types of words (e.g., orthographically related, phonologically related, semantically related). For example, it was found that morphological priming with pairs of words related by their roots [*hijo*/*hija* (son/daughter)] produced a sustained attenuation of the N400 during visual word recognition (Barber et al., 2002; Domínguez et al., 2004). In addition, Lavric et al. (2007) found that the mere appearance of a morphological relationship (“corner”—“CORN”) resulted in a robust reduction of the N400 event-related potential (ERP) component similar to that observed in pairs with genuine morphological relationships (“hunter”—‘HUNT’), supporting the existence of a purely structural morphemic segmentation procedure operating in the early stages of visual word recognition. Meanwhile, several functional magnetic resonance imaging (fMRI) studies employing a variety of tasks have provided evidence for distinct patterns of processing for regular and irregular past tense forms in English. More specifically, when directly compared to irregular inflection, the most consistently activated brain areas for regular inflection appear to be the left inferior frontal gyrus (LIFG) and its various subcomponents, often accompanied by basal ganglia and the cerebellum activity (Davis et al., 2004; Tyler et al., 2004, 2005; Joanisse and Seidenberg, 2005; Desai et al., 2006; Sahin et al., 2006; Bozic et al., 2010, 2015; Oh et al., 2011; Pliatsikas et al., 2014). For example, Bozic et al. (2007) reported a significant reduction in BOLD response in the LIFG for morphologically structured words in the visual modality. Arredondo et al. (2015) examined morphological awareness in the auditory modality in English-speaking children. Their results showed greater activation in the left frontal and temporoparietal junction when the children were asked to complete a morphological awareness task. In a recent MEG study using a primed lexical decision task, Cavalli et al. (2016) found evidence for a semantically driven morphological priming effect as early as 250 ms in left superior temporal gyrus (LSTG). Both orthographic and semantic contributions to morphological facilitation were found around 350 ms along the ventral stream and in the LIFG. Evidence for recombination of morphemes and semantic integration were found in the orbitofrontal cortex around 450–500 ms.

In addition to inflection and derivation in alphabetic languages, compounding has been considered an important morphological component of lexical productivity (Libben, 2014) because of the large number of novel compounds that can be created by compounding. Previous studies of compounding have found greater activation of the LIFG when the first part of a compound primed a picture, compared with unrelated primes (Koester and Schiller, 2011). Furthermore, Forgacs et al. (2012) found increased bilateral frontal and temporal activation for the processing of known compounds in German when compared to novel but phonologically valid compounds.

Unlike alphabetic languages in which affixes are orthographically joined to their stems, compounding is the primary word formation tool in Chinese (Wu et al., 2009) and is not orthographically marked. Chinese has many homographic morphemes that are indistinguishable using orthography or phonology alone (Zhou and Marslen-Wilson, 1995). More than 65% of 2423 Chinese characters in a database compiled by Liu et al. (2007) contained more than one meaning. For Chinese listeners, distinguishing homographic morphemes in spoken compound word recognition involves an interaction between the form and meaning of the constituent morphemes and also an interaction between the meanings of constituent morphemes and the whole words they compose. For example, the meaning of the syllable /kai1/(“
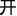
”) is different in different compound words. In /kai1 guan1/(“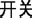,” “switch”), /kai1/ means open and /guan1/ means shut. However, in /kai1 shui3/(“
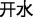
” “boiled water”), /kai1/ means boiled and /shui3/ means water. Therefore, the whole word provides contextual information that distinguishes different morpheme meanings when compound words are processed in the auditory modality. In a behavioral study using primed lexical decision tasks, Zhou and Marslen-Wilson (1999) found that words sharing common morphemes were facilitated in word recognition. Similar results were obtained in a word production study (Tsang et al., 2014) that found homographic morphemes to be interpreted with their more frequently used dominant meanings.

Although relatively few neuroimaging studies have been conducted on Chinese morphological processing, interesting and important results have been reported. Wu et al. (2017) in an ERP study using a primed visual lexical decision task found that compound words in which prime and target homographs were the same morpheme (e.g., the /gong1/ in /gong1 yuan2/ 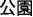
—public-garden “park” and /gong1 zhong4/ 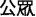
—public-people “the public”) produced a comparable P200 compared with prime and target homographs in which the prime and target homographs were different morphemes (e.g., /gong1 yuan2/ 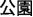
—public-garden “park” and 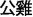
—male-chicken “rooster”). In contrast, N400 priming was identified only in the same-morpheme condition. Semantic sharing, in which the primes and targets were semantically related (e.g., /gong1 yuan2/ 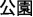
—public-garden “park” and /cao3 di4/ 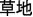
—grass-floor “lawn”) produced weaker effects. These findings indicate that homographic morphemes are activated during word recognition even though whole-word processing may be primary. Another study (Zou et al., 2015) found that the LIFG was strongly activated during an auditory morphological judgment task using compound pairs and that the LIFG activation was modulated by the degree of morphological relatedness between the two compounds. Liu et al. (2013) found that in making semantic relatedness judgments in Chinese compound words that were designed to either share identical morphemes or not, children with reading disabilities demonstrated less activation in the IFG than children with normal reading abilities. Following studies showing the importance of the IFG to morphological processing in Western languages, the Zou et al. (2015) and Liu et al. (2013) findings demonstrate the importance of the IFG in Chinese morphological processing.

The present study employed ERPs to investigate the neural underpinnings of Chinese spoken word morphological processing, and examined whether word-component morphological processing evokes neural activity that can be differentiated from that of whole-word semantic processing. In Zou et al. (2012), it was found that the phonology and orthography of the initial character of a Chinese compound significantly affected N400 amplitude. Significantly enhanced N400 amplitudes were found when an item was preceded by an orthographically dissimilar prime, and a delayed N400 was observed for word pairs sharing phonology. The phonology and orthography of the initial characters for the primes and targets were controlled in the present study to keep phonological and orthographic information constant.

In the lexical retrieval of Chinese compound words, the initial morpheme can in principle be accessed before the appearance of the second morpheme. But because Chinese is replete with homographic morphemes, the initial morpheme may be ambiguous, and so whole-word meaning is important because it plays a role in disambiguating the identity of the word-component homographic morphemes. However, in the processing of compound words, it is unclear whether the morphological processing of word-component morphemes is the same as whole-word semantic processing or whether it is decomposed and occurs as an independent process.

Previous studies have shown that the N400 is a stimulus-related brain activity in the 200–600 ms post-stimulus-onset window, typically examined in cross-condition comparisons and often appearing when comparing congruent and incongruent stimuli. The N400 effect routinely manifests as a monophasic negativity over centro-parietal sites that reflects differences in cognitive processing. The N400 is known to be sensitive to semantic integration processes in the case of semantic violations (Brown and Hagoort, 1999) and it is also observed for world knowledge violations, with an onset and peak latency that are similar in amplitude and distribution to the semantic N400 effect (Hagoort et al., 2004). In addition, most ERP studies on morphological processing using repetition priming find an N400 attenuation for morphologically related word pairs like *hunter-hunt* (Domínguez et al., 2004; Kielar and Joanisse, 2011; Lavric et al., 2011; Smolka et al., 2015; for a review, see Kutas and Federmeier, 2011; Leminen et al., 2018). However, when morphological and semantic processing are compared, some studies find no effect for semantic associations (e.g., Kielar and Joanisse, 2011) and some find N400 modulations for synonyms (Domínguez et al., 2004) or semantically associated verbs (Smolka et al., 2015), suggesting that the N400 is sensitive to semantic associations activated within a semantic network. Therefore, the present study addresses whether semantic integration and morphological processing are dissociated and might be expressed by differences in how the N400 is manifested.

For this purpose, we used an auditory-auditory priming paradigm (Zhou and Marslen-Wilson, 1995), manipulating the relationship between component morpheme and whole-word semantics to yield three prime-target word pair conditions: W+M+, W−M+, and W−M− (W = whole-word semantics, M = morpheme meaning, + = congruent, and − = incongruent). The first members of a prime-target pair always shared the same character, but the shared character did not always represent the same morpheme. When the first character of the prime-target pair represented the same morpheme, the value of “M” was “M+,” otherwise it was “M−.” When the semantics of the whole word were the same, the value of “W” was “W+,” otherwise it was “W−.” The ERPs were time-locked to the onset of the target words. Because the compound words were recorded as one complete audio file, when the subject hears the target word, cognitive processing begins from the initial syllable. The relationship between the first morpheme and whole word semantics has an effect on the recognition of the whole word. The second morpheme also provides information on whole word semantics, but since whole-word semantic information is already supplied by the first morpheme, therefore the effect of the second morpheme was not considered in the present study.

In the W+M+ condition, the first morphemes of the prime-target pairs were the same (M+) and they both matched the semantics of the whole word in which they appeared and the whole words were semantically related (W+), thereby resulting in no conflict between morpheme and whole-word semantics. For example, in the W+M+ prime-target pair /che1 lun2/ (car-wheel 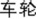 “wheel”) and /che1 tai1/ (car-tyre 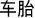 “tyre”), the first morpheme of the pairs *che1* was the same and therefore congruent (M+) and the two words /che1 lun2/ “wheel” and /che1tai1/ “tyre” are related (W+). In the second condition (W−M+), the first morpheme is the same (M+), but the whole word semantics of the prime and target are unrelated (W−). For example, the morpheme *huo3* “
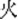
” in both /huo3 jian4/ (fire-arrow 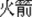 “rocket”) and /huo3 shan1/ (fire-hill 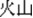 ‘volcano’) is the same and means “fire,” whereas the whole-word semantics of /huo3 shan1/ “volcano” and /huo3 jian4/ “rocket” are not related (W−). The third condition is W−M−, whose word pairs are not related in terms of either morphemic or whole-word semantics. For example, the homographic morphemes kai1 “
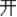
” in /kai1 guan1/ (open-shut 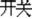 “switch”) meaning “open,” and kai1 “
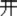
” in /kai1 shui3/ (boiled-water 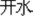 “boiled water”) meaning “boiled” are different (M−), and the two words “switch” “
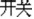
” and “boiled-water” “
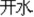
” in which they appear are also semantically unrelated (W−). Chinese is replete with homophones, and so distinguishing different morphemes is important during Chinese spoken word recognition. We hypothesized that the comparison between W−M+ vs. W−M− reflected participants’ sensitivity to the morphological status of word components in pairs of semantically unrelated compound words, which we defined as a morphological effect. In contrast, the comparison between W−M− vs. W+M+ was defined as the classic whole-word semantic effect. We hypothesized that the cognitive difference between whole-word and morphological processing, even in the auditory modality, would be manifested electrophysiologically in the form of different N400 profiles.

The participants were asked to perform an auditory lexical decision task, responding to target words that were preceded by primes. Our goal was to test whether morpheme and whole-word semantic relationships influence lexical decisions and whether differences in these relationships might be reflected in the time signature of ERP components. If morphological processing is distinct from whole word semantic processing, we posited that the classical semantic N400 effect would appear in the centro-posterior parts of the brain, and according to previous fMRI studies, the morphological N400 effect might appear in the anterior part of the brain. Conversely, if no ERP component differences are observed between whole-word and morphological processing, it may call into question whether morphological and whole-word processing are distinct during spoken word recognition.

In addition, research on child reading development has shown that morphological processing ability is equal or more important for reading in Chinese than in alphabetic languages (McBride-Chang et al., 2003; Shu et al., 2006; Wu et al., 2009; Ruan et al., 2018). Therefore, another purpose of the present study was to determine whether reading proficiency would affect morphological processing during spoken word recognition by examining the relationship between the neural activity of spoken language morphological processing and reading performance.

## Materials and Methods

### Participants

For the behavioral pilot experiment, 33 college students (mean age: 23 years, 17 female) were recruited from Beijing Normal University. Another 23 college students (mean age: 21.2 years, 11 females) participated in the ERP study. All participants were right-handed native speakers of Chinese with normal hearing and no reported neurological disorders. All participants gave written informed consent in accordance with the guidelines of the Human Subjects Committee of Beijing Normal University. Data from one participant in the behavioral experiment were discarded due to a high error rate [ER; i.e., greater than three standard deviations (SDs) from the group mean] and data from two participants in the ERP experiment were discarded due to excessive statistical artifact.

### Materials and Tests

A total of 270 disyllabic words were selected from a Chinese lexical database (Yu et al., 1998). All words were controlled by the phonology and orthography of the initial character, with the same phonology and orthography for the prime and target. The relationship between the morpheme meaning and whole-word semantics was manipulated and resulted in the three conditions W+M+, W−M+, and W−M−. The contrast between W+M+ and W−M− was regarded as a whole-word semantic effect, and the contrast between W−M+ and W−M− was regarded as a morphological effect. It was felt that these two contrasts would be critical in revealing the dissociation of morphological and whole-word semantic processing. Forty-five pairs of words were selected for each of the three conditions and matched for the following aspects of the first character of the prime and target: syllable frequency, phonological family size, whole word frequency, character frequency, number of strokes, and number of strokes in the second character (all *p*s > 0.57, see Supplementary Table S1).

**Figure 1:**
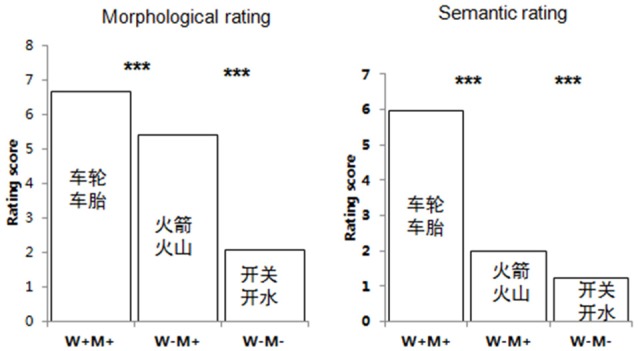
Semantic and morphological rating scores. Both semantic and morphological rating scores are significantly affected by the relationship between morpheme meaning and word semantics. ***p < 0.005.

The whole-word semantic relatedness and the relatedness of individual initial morphemes of each word pair were rated by two separate groups of 35 college students using a 7-point scale (1 = not related at all, and 7 = highly related). For the whole-word semantic rating, the participants were asked to judge the semantic relatedness of the whole-word pairs. A total of 434 semantically unrelated word pairs and 189 semantically related word pairs were randomly presented to the participants. Pairs with scores under 3 were defined as semantically unrelated, and pairs with scores above 4 were defined as semantically related. An additional group of 35 college students performed the morphological rating, in which they were asked to judge the semantic relatedness of the initial morphemes of the word pairs. The average whole word semantic rating scores were 5.94 (SD = 0.44) for W+M+, 1.76 (SD = 0.43) for W−M+, and 1.23 (SD = 0.23) for W−M−. A repeated measures analysis of variance (ANOVA) revealed significant main effects for semantic and morphological relatedness (all *p*s < 0.005). A *post hoc* analysis showed that the W+M+ score was significantly higher than both the W−M+ and W−M+ scores (all *p*s < 0.005). The whole-word semantic rating scores of W−M+ and W−M− were all below two, though the whole-word rating scores of the W−M+ pairs were rated significantly higher than the W−M− rating scores (*p* < 0.005), indicating that morphological information can affect whole-word semantics. Similarly, a *post hoc* analysis of the morphological rating scores found that the W+M+ and W−M+ rating scores were significantly higher than the W−M− scores (*p*s < 0.005), and that the W+M+ rating scores were significantly higher than the W−M+ scores (*p* < 0.005), suggesting that whole-word semantics can affect the meaning of individual morphemes. See Figure 1 and Table 1 for further details.

**Table 1:** The average scores for semantic and morphological rating.

	W+M+	W−M+	W−M−
Semantic rating (whole word)	5.95 (0.44)	1.97 (0.51)	1.22 (0.29)
Morphological rating (initial morpheme)	6.67 (0.21)	5.53 (0.61)	2.03 (0.41)

*Note: numbers in the parentheses is standard deviation*.

The concept of mutual information (MI) was used to define the extent of compound wordhood. The less word-like a given compound is, presumably the less likely participants are to process it *via* a whole-word storage route, and the more likely they are to pay attention to the individual morphemes in it. MI was collected on the website (Jun. Da. 2004. Chinese text computing^1^). The mean MI scores of primes and targets for each condition were: W+M+ prime, 5.65(SD = 2.35), W+M+ target, 5.26 (SD = 2.79), W−M+ prime, 4.99 (SD = 3.12), W−M+ target, 4.67 (SD = 3.07), W−M− prime, 3.99 (SD = 3.03), and W−M− target, 4.16 (SD = 2.85). A univariate ANOVA revealed that there was no significant main effect (*F*_(2,131)_ = 1.61, *p* = 0.21). These statistical data suggested that MI was unlikely to affect the main results and conclusion.

To investigate whether morpheme transparency is different between our experimental conditions, transparency ratings (Libben et al., 2003) for all the critical items were collected. We asked another 20 participants to rate the 270 compound words using a 7-point scale ranging from 1 to 7, where 1 = the meaning of the compound was not associated with the single morpheme, and 7 = the meaning of the compound was solely associated with the single morpheme. The first and second morphemes in the compound words were all rated, and average scores between the first and second ratings were used as the transparency index. The mean transparency scores of the second word for each pair of words were: W+M+, 5.00 (SD = 0.58), W−M+, 4.77 (SD = 0.86), W−M−, 4.66 (SD = 1.03). A repeated measures ANOVA showed that the main effect was not significant (*p* > 0.05), suggesting that the transparency factor was unlikely to affect our ERP results. On the other hand, transparency is indeed an important factor and should be given due attention in future studies on morphological processing.

For the lexical decision task, 240 pseudoword targets were created by changing the first or second character of a real word [e.g., changing the real word /zu3 zhuang1/ (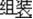) to the pseudoword /zu3 mei3/ (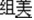)]. In both the real word and pseudoword trials, primes were always real words. Some of the word-pseudoword pairs shared phonology, and some did not, with the ratio being 1:2 for similar vs. dissimilar phonology (PseudoP+:PseudoP− = 1:2), thereby reducing the likelihood that participants would perform the lexical decision task using phonological similarity alone. The two pseudoword conditions (PseudoP+ and PseudoP−) were also matched for first syllable frequency, first syllable phonological family size, prime whole word frequency, prime first character frequency, prime first character stroke number, and prime second character stroke number (all *P*s > 0.05, see Supplementary Table S2).

All stimuli were recorded by a female native Mandarin speaker (with a standard Beijing accent) in a soundproof room on a digital audio recorder (Yamaha MG124C) using a CME MG-900 microphone at a sampling rate of 48 kHz with a 16-bit resolution. The mean duration of primes in each condition was 709 ms (SD = 35 ms) for W+M+, 716 ms (SD = 52 ms) for W−M+, 709 ms (SD = 42 ms) for W−M−, 713 ms (SD = 47 ms) for PseudoP+, and 716 ms (SD = 48 ms) for PseudoP−. The mean duration of targets in each condition was 710 ms (SD = 44 ms) for W+M+, 717 ms (SD = 51 ms) for W−M+, 705 ms (SD = 40 ms) for W−M−, 713 ms (SD = 48 ms) for PseudoP+, and 716 ms (SD = 46 ms) for PseudoP−. There was no significant difference in stimulus duration across conditions for either primes (*p* = 0.69 for words, *p* = 0.59 for pseudowords) or targets (*p* = 0.42 for words, *p* = 0.54 for pseudowords). The real words and pseudowords are listed in Supplementary Tables S3, S4.

### Basic Reading Tests

After finishing the electroencephalogram (EEG) session, the participants completed several reading tests to provide information about their reading skills. Three reading subtests were developed to gauge word reading skills: phonological extraction (onset judgment), orthographic processing (non-word cross-out task), and semantic integration (animal word cross-out task; Liu et al., 2017). A figure cross-out task was added as a baseline to control for general cognitive processing. Residuals of reading test scores were generated using the figure cross-out score as a regressor, with subsequent analyses conducted on the residuals. All tests were given a time limit to ensure that no participant could finish all items on any task. Several warm-up items were provided before each formal test. The reading scores were derived by subtracting the number of false responses from the number of correct responses.

### Figure Cross-Out Task

This task consisted of 100 instances of the figure § embedded in horizontal lines containing a total of 162 figures, such as 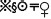. All figures were presented in random order on A4 paper, and the participants were asked to cross out as many of the § figures as possible in 30 s.

### Onset Judgment Task

This task required the participants to identify and mark single-character printed words containing a given onset. The stimuli consisted of 100 characters containing the onset /b/ (e.g., /bei3/, “
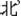
”) embedded in horizontal lines within a total of 308 high frequency single-character words with an average word frequency of 125 per million. The words were presented in random order on A4 paper, and the participants were asked to cross out as many words with the onset “b” as possible in 80 s.

### Non-character Cross-Out Task

Both real and non-characters (e.g., 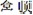) were included in this test. The non-characters were all combinations of actual components of Chinese characters. All the characters and non-characters were presented in random order. There were 162 items, 101 of which were non-characters. Participants were asked to cross out all the non-characters. The time limit for this task was 40 s.

### Animal Word Cross-Out Task

This task consisted of 220 two- and three-character words, including 74 animal words, such as “
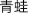
” (/qing1wa1/, “frog”). They were familiar words with an average word frequency of 16 per million. The animal and non-animal words were sequenced on A4 paper in a random order. The participants were asked to cross out all the animal words. The time limit for this task was 50 s.

### Procedure

#### Behavioral Experiment

The participants were tested individually in a quiet room. One trial consisted of the following sequence of events: a fixation point (+) was presented for 500 ms in the center of the screen; the spoken stimuli were presented binaurally through earphones while the fixation cross remained on the screen, with an interstimulus interval (ISI) of 150 ms between the prime and target. The participants were asked to respond as quickly and as accurately as possible after hearing the target word, indicating their response by pressing a button with their right hand if the target was a real word and with their left hand if it was a pseudoword. The participants were allotted 2,500 ms to indicate their response, with longer latencies being excluded from analysis. Between trials, a blank screen was presented for 1,000 ms. Reaction time (RT) was measured from the onset of the target stimulus until the participant pressed the response key. Items across different conditions were presented in a random order. Prior to completing the experimental trials, the participants were given 24 practice trials. Practice stimuli were items not included in the formal tests, and feedback was provided during the practice session.

#### ERP Experiment

The ERP experiment was identical to the behavioral task except for the inclusion of a delay after the presentation of a target item. The delay was incorporated into the ERP task to reduce the influence of decision and motion from waveforms associated with hand movement during lexical processing. Each trial consisted of the following sequence of events: a fixation point (+) was presented for 500 ms in the center of the screen, after which a spoken stimulus was presented while the fixation cross remained on the screen. A 150 ms ISI was included between the prime and target. After the target word appeared, the fixation cross remained on the screen for 1,000 ms, after which a question mark appeared. The participants were told to indicate their response when the question mark appeared on the screen. The question mark disappeared after the participant pressed the button, with a RT limit of 2,000 ms. To minimize artifacts due to eye blinks, participants were asked not to blink while the fixation cross was present. The inter-trial interval consisted of 1,000 ms of blank screen. A total of 24 practice trials were conducted before beginning the formal experimental trials.

### Data Recording and Analysis

#### Behavioral Data

Mean reaction times (RTs) and ERs for words and pseudowords were calculated separately for each condition (W+M+, W−M+, W−M−, PseudoP+, and PseudoP−). RTs greater than two SDs beyond the global mean were discarded (2.7%). Moreover, items with an ER above 50% across all subjects were excluded from the analysis (0.95%). This resulted in the exclusion of four items from the experiment (the percentage of items excluded for these reasons was 2.2% for words and 0.4% for pseudowords).

#### EEG Data

Continuous EEG data were recorded using a NeuroScan system at a sampling rate of 500 Hz using 64 Ag/AgCl electrodes, which was placed according to the extended 10–20 system. All scalp electrodes were referenced online to the left mastoid and were re-referenced offline to the average mastoid reference by subtracting one half of the activity recorded at the right mastoid. Vertical electrooculogram (VEOG) data were recorded from electrodes above and below the left eye, and horizontal electrooculogram (HEOG) data were recorded from electrodes placed at the outer canthi of both eyes. Impedances were kept below 5 kΩ. NeuroScan 4.5 system was used to analyze the ERP data. The data were filtered online using a band pass filter of 0.05–100 Hz, and off-line using a zero-phase shift digital filter (24 dB, bandpass signal: 0.05–30 Hz). Each trial was baseline corrected to the average voltage of the 200 ms pre-stimulus interval. Trials containing eye blinks and other artifacts were removed (determined by a maximum voltage criterion of ±70 μV on all scalp electrodes). Analyses were performed on the remaining trials (average non-rejected trials: 41/45 for W+M+, 40/45 for W−M+, 40/45 for W−M−, 73/89 for PseudoP+, and 145/151 for PseudoP−). ERPs were calculated from −200 to 1,000 ms and time locked to the onset of the target words. Combining previous morphological ERP studies (Domínguez et al., 2004; Kielar and Joanisse, 2011; Lavric et al., 2011; Smolka et al., 2015) and our own research (Zou et al., 2012), the mean N400 amplitudes were measured for 450 ms to 700 ms on nine scalp sites (FZ, CZ, PZ, F3, F4, C3, C4, P3, P4). Statistical analyses were conducted using SPSS16.0 (SPSS Inc, Chicago, IL, USA). For the midline analysis, a two-factor repeated measures ANOVA was conducted on the morphological conditions (W+M+, W−M+, and W−M−), with the anterior-posterior extent (anterior, middle, posterior) as factors. For the lateral analysis, a three-factorial repeated measures ANOVA was conducted using morphological conditions (W+M+, W−M+, and W−M−), laterality (left, right), and anterior-posterior extent (anterior, middle, posterior) as factors. We were particularly interested in the main effects of morphological conditions and the interaction of the morphological conditions by anterior-posterior extent. Other ancillary effects (e.g., main effects of laterality and anterior-posterior extent) are reported in the “Results” section, but are not further addressed in the “Discussion” section. Greenhouse-Geisser corrections (Geisser and Greenhouse, 1959) are applied where appropriate, with corrected *p*-values and original degrees of freedom reported.

## Results

### Behavioral Data

**Figure 2:**
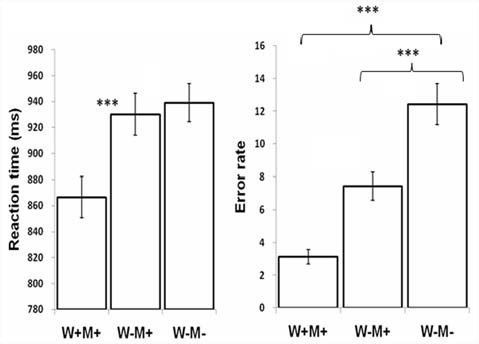
Reaction time (RT) and error rate (ER) during lexical judgment across different conditions. ***p < 0.005.

The reaction times (RT) and ERs across the W+M+, W−M+, and W−M− categories are displayed in Figure 2 and Table 2. The linear mixed-effects model (Baayen et al., 2008) was used to analyze the RT data using the R software (R Core Team, 2014). The results^2^ showed that a condition effect was significant (*F* = 17.65, *p* < 0.005). Contrasts showed that the RT for the W+M+ was significantly shorter than W−M− (*t* = −5.39, *p* < 0.005); no significant difference was found between W−M+ and W−M− (*t* = −0.628, *p* = 0.531). For the ERs, the generalized linear mixed-effects (logit) model (Jaeger, 2008) was used because the responses were binomial in nature. The results showed that the *F*-value for the condition effect was 10.50. Contrasts revealed the ERs for W−M− were significantly higher than both W+M+ (*z* = 4.52, *p* < 0.005) and W−M+ (*z* = 1.9, *p* = 0.05).

**Table 2:** Mean reaction time (RTs) and Error Rates (Err) in various priming conditions.

	Word			Pseudoword [-7pt]		W+M+	W−M+	W−M−	P+	P−
RTs (ms)	866.4 (87)	930.2 (90)	944 (95)	1,038 (136)	1,036 (134)
ER (%)	3.1 (2.4)	7.43 (4.7)	12.2 (5.8)	6.1 (4.9)	4.9 (3.9)

*Note: numbers in the parentheses is standard deviation*.

It should be noted that the relevant conditions were not completely matched on whole-word relatedness or initial-morpheme relatedness. For example, the morphologically related condition (W−M+) and unrelated condition (W−M−) should presumably be equally low in whole-word relatedness (i.e., the pattern should be W+M+ > W−M+ = W−M−); however, this was not the case. Likewise, W−M+ and W+M+ should presumably be equally high in initial-morpheme relatedness (the pattern should be W+M+ = W−M+ > W−M−), but this was not the case either. This makes it hard to attribute differences to just morpheme or whole-word relatedness since these factors differed even in comparisons where they were not meant to be manipulated. Therefore, the next step was to test the effects of whole word semantics and morphology on RT and ERs, respectively. For whole word semantic effect, one-way ANOVA was conducted between W+M+ and W−M+ and whole word semantic rating scores were entered as a covariate. Results showed that whole word semantics significantly predicated the RT difference between W+M+ and W−M+ (*F*_(1,87)_ = 4.08, *p* = 0.003). For morphological effect, one-way ANOVA was conducted between W−M+ and W−M− and morphological rating scores were entered as a covariate. Results showed that morphological states significantly predicted the ER difference between W−M+ and W−M− (*F*_(1,87)_ = 8.724, *p* = 0.004).

For pseudoword pairs, RT analysis showed no significant difference between PseudoP+ and PseudoP− (*t*_(31)_ = 0.49, *p* > 0.05). The ER analysis showed a marginally significant difference, with more errors for PseudoP+ than PseudoP− (*t*_(31)_ = −1.76, *p* = 0.09).

Because a response-delay paradigm was applied during the ERP data collection, the RTs and ERs reported above came from the behavioral experiment. In the ERP experiment, the average correct ratio was very high, with 97.7% for the W+M+, 96.3% for the W−M+, and 95.6% for the W−M−, with no significant difference across the three conditions. These results indicate that participants performed adequately and that there were no significant differences in task difficulty in the ERP experiment.

### ERP Data

#### N400 Amplitude

**Figure 3:**
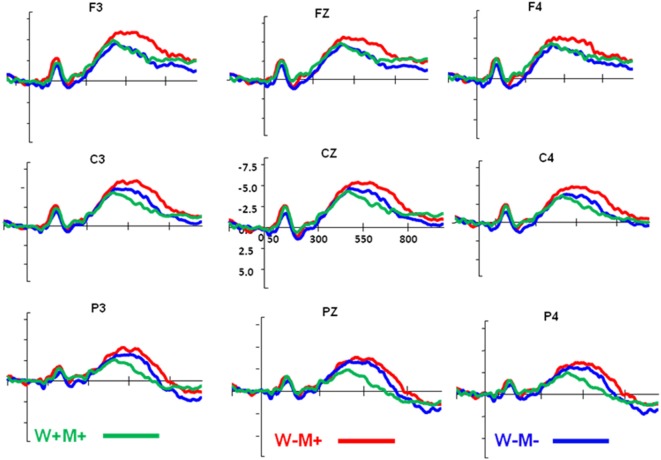
Event-related potentials (ERPs) across different priming conditions from the nine representative electrodes. The green line represents the W+M+ condition, the red line represents the W−M+ condition, and the blue line represents the W−M− condition. The unit of vertical coordinate is μV.

Overall, we observed that the amplitude of the N400 was modulated by morphological and semantic information (Figure 3). The ANOVA on the midline region showed a significant main effect for the morphological condition (*F*_(2,40)_ = 14.41, *p* < 0.005) and anterior-posterior location (*F*_(2,40)_ = 31.63, *p* < 0.005). The morphological condition for anterior-posterior interaction was also significant (*F*_(4,80)_ = 5.45, *p* = 0.001). Since we were more interested in morphological and whole word semantic effects, planned comparisons were conducted on FZ and PZ separately. For FZ, the comparison was based on the contrast of W−M+ vs. W−M−. It was found that the N400 amplitude was significantly increased for W−M+ relative to W−M− (*t*_(60)_ = −2.53, *p* = 0.014, two-tailed), suggesting that the morphological effect appeared when the morpheme meaning and whole word semantics were incongruent. For PZ, the comparison was based on the contrast of W+M+ vs. W−M−. The results showed that a classic semantic N400 was elicited with significant decreased N400 amplitude for W+M+, as compared to W−M− (*t*_(60)_ = 3.9, *p* < 0.005).

The results of the lateral region ANOVA showed significant main effects for the morphological condition (*F*_(2,40)_ = 8.22, *p* < 0.001), laterality (*F*_(1,20)_ = 4.36, *p* = 0.05), and anterior-posterior extent (*F*_(2,40)_ = 19.66, *p* < 0.005). There was no three-way interaction found for the morphological condition, laterality, and anterior-posterior extent. Overall, the patterns of the three morphological conditions in the left and right hemispheres were similar to the midline analysis. The main effect of laterality was significant, and because we were more interested in the interaction of the morphological and anterior-posterior conditions, results for the morphological conditions for the left and right regions were analyzed separately. For the left hemisphere, the ANOVA revealed significant main effects for the morphological condition (*F*_(2,40)_ = 18.84, *p* < 0.005), anterior-posterior extent (*F*_(2,40)_ = 30.24, *p* < 0.005), and a significant morphological/anterior-posterior interaction (*F*_(4,80)_ = 7.53, *p* < 0.005). Because we were more interested in morphological and whole word semantic effects, planned comparisons between these two effects were conducted separately. We found that the amplitude of the N400 was significantly enhanced for W−M+ relative to W−M− on the F3 (*t*_(60)_ = −2.85, *p* = 0.006), and a classic semantic N400 was elicited for the W+M+ as compared to the W−M− on P3 (*t*_(60)_ = 3.86, *p* < 0.005). For the right hemisphere, the ANOVA found a significant main effect only for anterior-posterior extent (*F*_(2,40)_ = 10.28, *p* < 0.005), with no main effect for morphological condition (*F*_(2,40)_ = 1.54, *p* = 0.23), and no morphological conditions by anterior-posterior interaction (*F*_(4,80)_ = 1.02, *p* = 0.41). The lateral analysis indicated that the dissociation between the morphological and semantic processing mainly occurred in the left and central scalp regions.

### Topographical Analysis

**Figure 4:**
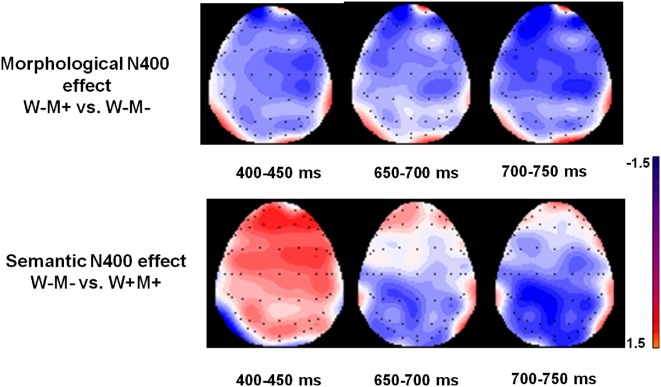
The voltage maps of the morphological N400 effect and semantic N400 effect. The topographic map of morphological N400 effect is based on the differences in waves between the W−M+ and W−M− conditions. The topographic map of the semantic N400 effect is based on the differences in waves between the W−M− and W+M+ conditions.

Topographical analyses using average amplitude differences, running in the 450–700 ms time window, revealed different topographies for the morphological and whole-word semantic effects (Figure 4). The morphological effect was obtained by subtracting the W−M− from the W−M+, and the whole-word semantic effect was obtained by subtracting the W+M+ from the W−M−. As shown in Figure 4, the morphological N400 effect was largely distributed in the left anterior and central anterior scalp regions. In contrast, the whole-word semantic N400 effect was mainly distributed in the left posterior and central posterior scalp regions.

### Correlation Between Morphological N400 Effect and Basic Reading Performance

**Figure 5:**
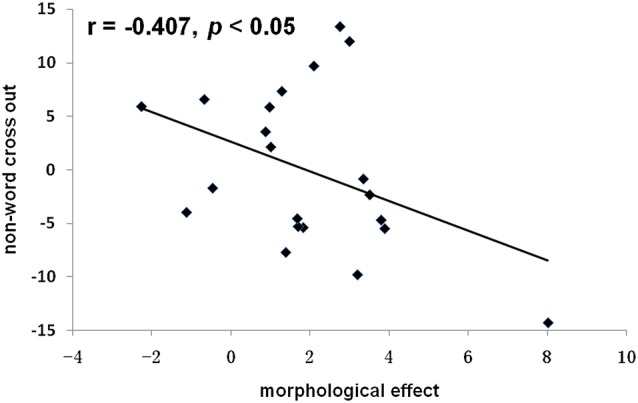
Correlations between the morphological N400 effect and non-word cross out (residual). The morphological N400 effect was calculated as the difference of the mean N400 amplitude between the W−M+ and W−M− conditions (on FZ electrode). r stands the Pearson correlation coefficient.

We transformed the reading scores into the number of correct items read per minute. The average reading score for onset judgment was 27.18 (SD = 6.31), non-word cross-out score was 58.9 (SD = 9.19), and animal word cross-out score was 43.20 (SD = 6.85). A Pearson correlation analysis was conducted for the morphological N400 effect and the behavioral performance of the three reading tasks (residuals after controlling for figure cross out). We found a significant negative correlation between the morphological N400 effect and the non-word cross out: *r* = −0.407, *p* < 0.05 (Figure 5). The correlation between the morphological N400 effect and the onset judgment (*r* = 0.03, *p* > 0.05), and the animal word cross out (*r* = 0.04, *p* > 0.05) did not reach a level of statistical significance.

## Discussion

Recent years, the neural basis of morphological processing was mostly explored in the visual modality, few research has been reported in auditory modality. Unlike English and other Western languages in which morphologically complex words contain inflectional and derivational affixes (Crepaldi et al., 2016), there is no morphological marker on the morphemes of Chinese compound words. In Chinese, polysemous morphemes are prevalent in compound words. A given character sharing the same phonology and orthography might correspond to several different morphemes. For Chinese listeners, distinguishing homographic morphemes in spoken compound word recognition involves an interaction between the form and meaning of the constituent morphemes and also an interaction between the meanings of constituent morphemes and the whole words they compose. However, up to date, in the processing of Chinese compound words, it is unclear whether the morphological processing of word-component morphemes is the same as whole-word semantic processing or whether it is decomposed and occurs as an independent process. For this purpose, we used an auditory-auditory priming paradigm (Zhou and Marslen-Wilson, 1995), manipulating the relationship between morphemic and whole-word semantics to yield the three prime-target word pair conditions of W+M+, W−M+, and W−M−. The phonology and orthography of the initial characters of the primes and targets were controlled to hold phonological and orthographic information constant. This is the first study on the neural basis of Chinese morphological processing in auditory modality.

Behaviorally, both RT and ER results showed that if pairs shared morphemic information (M+), spoken word recognition was facilitated, with shorter RT and lower ER for W+M+ and W−M+ relative to W−M−. These results are consistent with previous studies demonstrating that morphological processing is an independent factor in reading and listening (Zhou and Marslen-Wilson, 1995, 1999; Domínguez et al., 2004; Bozic et al., 2007; Bick et al., 2008, 2010; Marslen-Wilson et al., 2008; Tyler and Marslen-Wilson, 2008; Tsang and Chen, 2013). Even though the whole word rating scores for W−M+ were significantly higher than those for W−M−, the fact that the mean rating for both W−M+ (*M* = 1.76) and W−M− (*M* = 1.23) was very low, suggesting they are semantically unrelated. The ER for W−M+ was significantly lower than those for W−M−, suggesting morphological information played an important role during Chinese spoken word recognition.

However, because morpheme and whole-word semantics are closely related in complex word formation, it is worth noting the average rating score for both W−M+ and W−M− was very low though the whole-word rating scores for W−M+ were significantly higher than those for W−M−. Therefore, it is hard to absolutely separate semantic and morphological processing in Chinese compound words, new paradigm and technology are needed to disentangle them more precisely in the future.

The ERP data revealed that the amplitude of the N400 was clearly differentially modulated by morphological and whole-word semantic information and that there was a topographical dissociation between them. Specifically, the morphological effect elicited a significant enhanced N400 (ranging from about 400 ms to 700 ms) at the left anterior part of the brain (e.g., F3, FZ), while the whole-word semantic condition (W+M+) elicited a classic semantic N400 effect at the left central-posterior part of the brain (i.e., P3, PZ). Previous studies have also tried to separate the different neural basis of morphology and semantics. Beyersmann et al. (2014) conducted a primed visual lexical decision study comparing a morphological [LAVAGE—laver (washing—wash)], a semantic [LINGE—laver (laundry—wash)], an orthographic [LAVANDE—laver (lavender—wash)], and an unrelated control condition [HOSPICE—laver (nursing home—wash)]. They found that semantic priming arises later than morphological priming; specifically, significant morphological but no semantic priming at 100–250 ms was revealed. Beyersmann et al. (2014) and most other previous morphological studies (Domínguez et al., 2004; Kielar and Joanisse, 2011; Lavric et al., 2011; Smolka et al., 2015) were conducted in visual modality, early visual processing was important and was expressed in the ERP data. The initial phonology and orthography of the first syllable were controlled completely consistent in the present study, the ERP wave difference appeared rather late and hardly the same time window. A pure difference between morphological and semantic effect in the compound spoken words was revealed in our experiment. To the best of our knowledge, the present study is the first to report the topographical dissociation of word-component morphological processing from whole word semantic processing in Chinese spoken word recognition.

Concerning the results of the behavioral experiment, the RT was shorter for W−M+ relative to W−M− and the whole-word semantic rating scores were higher. If behavioral facilitation for W−M+ is attributed to the relatively higher rating scores on whole-word semantics rather than sharing morpheme meaning, it would be reasonable to observe a reduced N400 for the W−M+ due to semantic priming than the W−M−. However, the results showed that the amplitude of the W−M+ was enhanced rather than reduced relative to the W−M−. Therefore, we inferred that the modulation of the N400 in the anterior part of the brain was motivated by morphology rather than whole-word semantics. One might question why sharing a morpheme shortened the RT while enhancing the amplitude of 400. We believe that the behavioral data reflected the final state of cognitive processing as compared with the ERPs which would show the on-line real-time course of processing. For the W−M+, although prime and target were unrelated for word semantics, they shared the same morpheme meaning of the first syllable. The complex relation between morpheme meaning and word meaning in the W−M+ produced a morpheme-word semantic conflict that made it difficult to access the word, so the enhanced N400 may reflect the conflict resolution for the integration of morpheme and word meaning during spoken word recognition. As for the classic semantic N400 effect, this was consistent with previous results showing that semantically related conditions significantly reduced the amplitude of N400 in the central-posterior part of the brain (Kutas and Hillyard, 1984; Connolly et al., 1995). These data provided clear evidence for the dissociation of morphological and whole-word semantic processing during Chinese spoken word recognition, lending further support to the independent role of Chinese morphological processing in lexical access (Zhou and Marslen-Wilson, 1995, 1999; Tsang and Chen, 2013).

The usual way of constructing morphologically complex words in alphabetic languages is to use affixation, and it has been proposed that a purely structural morphemic segmentation could occur in the early stages of visual word recognition. There is broad consensus that the visual word recognition system is sensitive to morphological structure (e.g., hunter = hunt + er), and this conclusion has also been supported by ERP studies. For instance, Domínguez et al. (2004) explored the attributes of ERPs evoked by morphological, homographic, orthographic, and semantic priming, and found that morphological priming (related by stem) produced a sustained attenuation of N400. Meanwhile, homographic priming (with a superficially similar stem, but without morphological or semantic relation) produced an initial attenuation similar to the morphological pairs, then tended to form a delayed N400 due to the impossibility of integration. The orthographic priming produced an effect similar to that of unrelated pairs, and the synonyms (semantic priming) advanced more slowly than morphological pairs, finally producing a more positive peak around 600 ms. One important theoretical comment is that, irrespective of how morphological decomposition is conceptualized, morphologically complex words share representations with their stems only in cases in which there is a semantic relationship between them (Plaut and Gonnerman, 2000; Davis et al., 2004). Therefore, morphological effects are typically expected in cases in which a morphologically complex word and its stem are semantically related (Lavric et al., 2007). However, there is no explicit orthographic marking of morphological affixation in Chinese to aid in the dissociation of morphemes in compound words because the two characters of such compounds have the same spacing as any two adjacent characters and because their morphemic meaning is usually strongly connected with word semantics. In consideration of this distinctive characteristic of Chinese, we tried to manipulate the relationship between morpheme meaning and word semantics, which has not been investigated in previous behavioral and ERP studies. Combining both the behavioral and ERP data, we conclude that Chinese word-component morphological processing is independent of whole-word semantics, showing a dissociated neural pattern between them, which was further supported by our topographical analysis results. More specially, a left frontal-anterior distribution and an enhanced amplitude of N400 were produced by morphologically-related pairs. The topographical distribution was consistent with previous findings that the LIFG is the principal brain area in which Chinese morphological judgment occurs. On the other hand, a classic semantic N400 in the central-posterior part of the brain was found for semantically related word pairs. The enhanced N400 for the morphologically related condition was interpreted as a reflection of the processing conflict that occurred while participants tried to integrate morphemic and whole-word semantics in W−M+.

The behavioral results relating Chinese morphological processing and basic reading skills lend support to previous findings that morphological awareness—defined as awareness of and access to morphemes in words—is critically important in Chinese reading development (Shu et al., 2006). As children learn to read Chinese, they implicitly seek systematic associations and regularities between spoken and written language. Because Chinese has many homophones, learning to distinguish them in spoken language may be associated with character recognition ability (McBride-Chang et al., 2003). Therefore, an additional aim of the present study was to explore to what extent reading proficiency affects morphological processing during spoken word recognition. It was found that the morphological N400 effect was negatively correlated with non-word cross out, while no correlation was found between the morphological N400 effect and performance in the onset judgment or animal word cross-out tasks. The onset judgment, non-word cross-out and animal word cross-out tasks reflect three important components of reading: phonological abstraction, orthographic processing, and semantic integration. The morphological N400 effect was found to be negatively correlated with orthographic processing rather than the other two components, suggesting that participants with higher reading ability relied less on visual orthographic information in resolving the incongruence between morpheme meaning and whole word semantics during spoken word processing. To our knowledge, this is the first study providing evidence of the relationship between reading skills and the neural basis of morphological processing during spoken word recognition. How the morphological N400 effect interacts with reading ability in normal readers and readers with reading disabilities is an important area for future investigations.

In conclusion, the present study provides electrophysiological evidence of the dissociation of morphological and whole-word semantic processing in Chinese spoken word processing. The results identified a central-anterior morphological N400 effect when morphemic meaning conflicts with whole-word semantics, in addition to a classic semantic N400 effect with significantly reduced amplitude in central-parietal areas. Our results also showed that the morphological N400 effect is negatively correlated with reading ability. These results demonstrate the important role of morphological processing in Chinese spoken word recognition and support the notion that morphological analysis is an important component of reading skill development in Chinese.

## Ethics Statement

This study was carried out in accordance with the recommendations of “Ethics Committee of Beijing Normal University” with written informed consent from all subjects. All subjects gave written informed consent in accordance with the Declaration of Helsinki. The protocol was approved by the “Ethics Committee of Beijing Normal University.”

## Author Contributions

LZ, YL and HS designed the research. LZ and ZX collected and analyzed the data. LZ, JP, ZX, and HS wrote the article.

## Conflict of Interest Statement

The authors declare that the research was conducted in the absence of any commercial or financial relationships that could be construed as a potential conflict of interest.

## Footnotes

^1^http://lingua.mtsu.edu/chinese-computing/

^2^These were consistent with the results obtained using a traditional ANOVA, which are provided in the Supplementary Material.
