# Supplementary material for: Morphological and Whole-Word Semantic Processing Are Distinct: Event Related Potentials Evidence From Spoken Word Recognition in Chinese
Source: Front Hum Neurosci. 2019 Apr 17;13:133. doi: 10.3389/fnhum.2019.00133 (PMC6478770; doi:10.3389/fnhum.2019.00133)
Supplement: Supplementary file 1 [file Table_1.DOC]

**Supplementary Material**

Supplementary Material

**Morphological and Whole-Word Semantic Processing Are Distinct: Event Related Potentials Evidence from Spoken Word Recognition in Chinese**

**Lijuan Zou^a^,** **Jerome L. Packard^b^, Zhichao Xia^c^, Youyi Liu^c^, and Hua Shu^c*^**

^a^School of Psychology and Education, Zaozhuang University, China

^b^Beckman Institute, University of Illinois, Urbana, Illinois, USA

^c^State Key Laboratory of Cognitive Neuroscience and Learning & IDG/McGovern Institute for Brain Research, Beijing Normal University, China; Center for Collaboration and Innovation in Brain and Learning Sciences, Beijing Normal University, China

*Corresponding Author ([**shuhua@bnu.edu.cn**](mailto:shuhua@bnu.edu.cn))

**Traditional ANOVA analysis on behavioral data**

The response times (RT) and error rates across the W+M+, W-M+, and W-M- categories are shown in Table 2 and Figure 2. For RTs, a repeated-meansures ANOVA showed a significant main effect in both subject and item analysis (F1(2, 62) = 118.95, p < 0.005; F2 (2, 82) = 33.62, p < 0.005). The post hoc analysis on the subject data shows that the RT for the W+M+ was significantly shorter than both the W-M+ (t31 = -13.59, p < 0.005) and the W-M- (t31 = -12.27, p < 0.005), and a marginally significant difference between the W-M+ and the W-M- (t31 = -1.9, p = 0.06). The analysis of error rate found a significant main effect in both the subject and items analysis (F1(2, 62) = 34.93, p < 0.005; F2 (2, 82) = 13.56, p < 0.005). The post hoc analysis on the subject data shows that the error rate for the W+M+ items was significantly less than both the W-M+ (t31 = -4.66, p < 0.005) and the W-M- (t31 = -7.25, p < 0.005) items, with the error rate for the W-M+ items significantly less than for the W-M- (t31 = -4.52, p < 0.005) items.

# Figures legends

**Figure 1.** Semantic and morphological rating scores. Both semantic and morphological rating scores are significantly affected by the relationship between morpheme meaning and word semantics. ***, *p* < 0.005.

**Figure 2.** Reaction time (RT) and error rate during lexical judgment across different conditions. Error bar = S.E.M. ***, *p* < 0.005; +, marginally significant.

**Figure 3.** Event-related potentials across different priming conditions from the nine representative electrodes. The green line represents the W+M+ condition, the red line represents the W-M+ condition, and the blue line represents the W-M- condition. The unit of vertical coordinate is μV.

**Figure 4.** The voltage maps of the morphological N400 effect and semantic N400 effect. The topographic map of morphological N400 effect is based on the differences in waves between the W-M+ and W-M- conditions. The topographic map of the semantic N400 effect is based on the differences in waves between the W-M- and W+M+ conditions.

**Figure 5.** Correlations between the morphological N400 effect and non-word cross out (residual). The morphological N400 effect was calculated as the difference of the mean N400 amplitude between the W-M+ and W-M- conditions (on FZ electrode). r stands the Pearson correlation coefficient.

# Tables

Table 1. The average scores for semantic and morphological rating

|  | W+M+ | W-M+ | W-M- |
| --- | --- | --- | --- |
| Semantic rating (whole word) | 5.95 (0.44) | 1.97 (0.51) | 1.22(0.29) |
| Morphological rating (initial morpheme) | 6.67(0.21) | 5.53(0.61) | 2.03(0.41) |

Note: Numbers in the parentheses is standard deviation.

Table 2. Mean reaction time (RTs) and Error Rates (Err) in various priming conditions

|  |  | Word |  | Pseudoword | |
| --- | --- | --- | --- | --- | --- |
|  | W+M+ | W-M+ | W-M- | P+ | P- |
| RTs(ms) | 866.4(87) | 930.2(90) | 944(95) | 1038(136) | 1036(134) |
| Err(%) | 3.1(2.4) | 7.43(4.7) | 12.2(5.8) | 6.1(4.9) | 4.9(3.9) |

Note: Numbers in the parentheses is standard deviation.

# Supplementary Tables

**Supplementary table 1. Characteristics of real words**

| TYPE | Example | SRS | MRS | Prime  FSF | Target  FSF | Prime  FCPF | Target  FCPF | Prime  WF | Target  WF | Prime  FCF | Target  FCF | Prime  FNS | Target  FNS | Prime  SNS | Target  SNS | PD  (ms) | TD  (ms) |
| --- | --- | --- | --- | --- | --- | --- | --- | --- | --- | --- | --- | --- | --- | --- | --- | --- | --- |
| W+M+ | 车轮(wheel)  车胎(tyre) | 5.94  (0.44) | 6.67  (0.21) | 289.91  (578.08) | 289.91  (578.08) | 5.31  (3.32) | 5.31  (3.32) | 12.06  （14.04） | 16.36  (61.07) | 129.12  (473.72) | 129.12  (473.72) | 7.57  (2.75) | 7.57  (2.75) | 8.44  (2.54) | 8.02  (2.71) | 709  (35) | 710  (44) |
| W-M+ | 火山(volcano)  火箭(rocket) | 1.76  (0.43) | 5.35  (0.67) | 232.14  (285.14) | 232.14  (285.14) | 4.43  (2.55) | 4.73  (2.55) | 14.20  （44.58） | 13.20  (19.71) | 152.21  (256.09) | 152.21  (256.09) | 7.04  (2.75) | 7.04  (2.75) | 8.62  (3.52) | 7.97  (2.97) | 716  (52) | 717  (51) |
| W-M- | 开水(boiled water)  开关(switch) | 1.23  (0.33) | 1.99  (0.37) | 258.13  (435.78) | 258.13  (435.78) | 5.35  (2.87) | 5.35  (2.87) | 12.19  （20.92） | 11.54  (18.76) | 111.88  (186.11) | 111.87  (186.11) | 7.57  (2.65) | 7.57  (2.65) | 8.11  (3.39) | 7.91  (3.42) | 709  (42) | 705  (40) |
| F |  | 1812.73 | 1250.46 | 0.18 | 0.19 | 0.37 | 0.78 | 0.07 | 0.18 | 0.17 | 0.17 | 0.56 | 0.56 | 0.29 | 0.02 | 0.37 | 0.87 |
| *p* |  | <0.005 | <0.005 | 0.83 | 0.83 | 0.94 | 0.62 | 0.93 | 0.84 | 0.84 | 0.84 | 0.57 | 0.57 | 0.74 | 0.98 | 0.69 | 0.42 |

Note: SRS: semantic related scores; MRS: morphological related scores; FSF: the frequency of the first syllabler per million; FCPF: the phonological family size of first syllable; WF: whole word frequency per million; FCF: the frequency of first character per million; FNS: the number of strokes of first character; SNS: the number of strokes of second character; PD: prime duration; TD: target duration.The number in the parenthesis is standard deviant. These data were collected from the Chinese lexical database (Yu et al., 1998).

**Supplementary table 2. Characteristics of pseudoword**

| TYPE | Example | Prime FSF | Target FSF | Prime FCPF | Target FCPF | Prime  WF | Prime FCF | Prime FNS | Prime SNS |
| --- | --- | --- | --- | --- | --- | --- | --- | --- | --- |
| P+ | 毛巾(/mao2/)  毛麦(/mao2/) | 386.905  (610.898) | 386.905  (610.898) | 5.549  (3.084) | 5.549  (3.084) | 10.608  (19.168) | 163.138  (328.853) | 7.878  (3.294) | 9.012  (3.636) |
| P- | 底座(/di3/)  报脑(/bao4/) | 421.679  (871.578) | 365.485  (847.346) | 5.384  (4.084) | 5.899  (3.834) | 9.364  (24.297) | 146.810  (378.918) | 8.208  (3.019) | 8.145  (3.064) |
| t |  | -0.323 | 0.203 | 0.322 | -0.717 | 0.403 | 0.331 | -0.778 | 1.952 |
| *p* |  | 0.747 | 0.839 | 0.748 | 0.474 | 0.687 | 0.741 | 0.437 | 0.052 |

Note: FSF: the frequency of the first syllabler per million; FCPF: the phonological family size of first syllable; WF: whole word frequency per million; FCF: the frequency

of first character per million; FNS: the number of strokes of first character; SNS: the number of strokes of second character. The number in the parenthesis is standard deviant. These data were collected from the

Chinese lexical database (Yu et al., 1998).

**Supplementary table 3. Materials used in behavioral and ERP experiments – Word conditions**

| **W+M+** | | **W-M+** | | **W-M-** | |
| --- | --- | --- | --- | --- | --- |
| prime | target | prime | target | Prime | target |
| 外语 | 外文 | 高空 | 高温 | 被褥 | 被害 |
| 夜晚 | 夜间 | 白发 | 白鸽 | 立功 | 立刻 |
| 冬季 | 冬天 | 伴侣 | 伴奏 | 面包 | 面孔 |
| 波浪 | 波涛 | 温室 | 温差 | 导师 | 导体 |
| 高唱 | 高歌 | 半年 | 半径 | 开关 | 开水 |
| 剧场 | 剧院 | 定律 | 定期 | 开车 | 开除 |
| 店主 | 店家 | 悲观 | 悲壮 | 资格 | 资助 |
| 景色 | 景致 | 边关 | 边际 | 节奏 | 节水 |
| 泪珠 | 泪花 | 大风 | 大门 | 景物 | 景仰 |
| 晚餐 | 晚饭 | 红茶 | 红星 | 钟头 | 钟爱 |
| 车轮 | 车胎 | 补丁 | 补助 | 面具 | 面条 |
| 演讲 | 演说 | 同期 | 同胞 | 运费 | 运气 |
| 邮政 | 邮递 | 毛笔 | 毛驴 | 注册 | 注重 |
| 索取 | 索要 | 内心 | 内战 | 故乡 | 故障 |
| 课堂 | 课程 | 真相 | 真心 | 剧痛 | 剧组 |
| 药品 | 药物 | 火箭 | 火山 | 来回 | 来年 |
| 秋季 | 秋天 | 乌鸦 | 乌云 | 表皮 | 表哥 |
| 描写 | 描绘 | 巨浪 | 巨人 | 理发 | 理会 |
| 气候 | 气象 | 水果 | 水库 | 节能 | 节拍 |
| 光亮 | 光线 | 聚餐 | 聚焦 | 院落 | 院士 |
| 风暴 | 风浪 | 本土 | 本职 | 快餐 | 快乐 |
| 月亮 | 月球 | 保温 | 保养 | 旅店 | 旅长 |
| 任命 | 任用 | 绿洲 | 绿豆 | 长相 | 长辈 |
| 山峰 | 山脉 | 宝库 | 宝座 | 局长 | 局限 |
| 乡村 | 乡下 | 特权 | 特性 | 阵雨 | 阵势 |
| 旅店 | 旅馆 | 双臂 | 双目 | 照料 | 照搬 |
| 买方 | 买主 | 晚年 | 晚霞 | 气色 | 气垫 |
| 废品 | 废物 | 全文 | 全军 | 解雇 | 解说 |
| 退还 | 退回 | 黑板 | 黑人 | 情报 | 情操 |
| 诚挚 | 诚恳 | 积雪 | 积蓄 | 明年 | 明星 |
| 火光 | 火焰 | 同上 | 同行 | 机会 | 机械 |
| 温柔 | 温和 | 背景 | 背影 | 庄园 | 庄重 |
| 花圃 | 花园 | 平房 | 平头 | 草稿 | 草帽 |
| 山地 | 山岭 | 黄豆 | 黄金 | 分子 | 分区 |
| 节约 | 节省 | 彩旗 | 彩虹 | 纵容 | 纵向 |
| 喜事 | 喜讯 | 阶梯 | 阶层 | 草丛 | 草案 |
| 西部 | 西边 | 深山 | 深秋 | 机场 | 机遇 |
| 强迫 | 强制 | 话题 | 话筒 | 信箱 | 信仰 |
| 生存 | 生活 | 按摩 | 按钮 | 花费 | 花灯 |
| 阻力 | 阻挡 | 阴影 | 阴雨 | 号召 | 号码 |
| 查阅 | 查找 | 手枪 | 手套 | 天线 | 天才 |
| 讲课 | 讲解 | 花纹 | 花粉 | 服药 | 服气 |
| 伤病 | 伤口 | 手语 | 手绢 | 值钱 | 值班 |
| 戏剧 | 戏曲 | 废除 | 废铁 | 生计 | 生病 |
| 黄沙 | 黄土 | 新闻 | 新年 | 空想 | 空军 |

**Supplementary table 4. Materials used in behavioral and ERP experiments – Pseudoword conditions**

| **Pseudo P+** | | | | | | | | | | | | | | |  |
| --- | --- | --- | --- | --- | --- | --- | --- | --- | --- | --- | --- | --- | --- | --- | --- |
| prime | target | | prime | | target | | prime | | target | | prime | | target | |  |
| 堤坝 | 堤渣 | | 初级 | | 初易 | | 开创 | | 开兰 | | 粗细 | | 粗暖 | |  |
| 观看 | 观钉 | | 正规 | | 正哲 | | 本性 | | 本雄 | | 组装 | | 组每 | |  |
| 博览 | 博驹 | | 浪潮 | | 浪秩 | | 值得 | | 值乱 | | 双杠 | | 双尝 | |  |
| 波纹 | 波东 | | 后来 | | 后汕 | | 机枪 | | 机择 | | 线索 | | 线渴 | |  |
| 理解 | 理升 | | 气压 | | 气浊 | | 沉沦 | | 沉今 | | 时光 | | 时皮 | |  |
| 德语 | 德甘 | | 饭桌 | | 饭缔 | | 注射 | | 注凑 | | 文摘 | | 文起 | |  |
| 晚会 | 晚兜 | | 乘凉 | | 乘含 | | 案件 | | 案染 | | 微弱 | | 微土 | |  |
| 钟摆 | 钟弯 | | 医生 | | 医努 | | 海豚 | | 海捎 | | 表扬 | | 表宠 | |  |
| 容忍 | 容咕 | | 热忱 | | 热静 | | 毛巾 | | 毛麦 | | 乡土 | | 乡狂 | |  |
| 人品 | 人淞 | | 长短 | | 长仓 | | 快艇 | | 快峦 | | 桥墩 | | 桥患 | |  |
| 比方 | 比孪 | | 财主 | | 财媚 | | 废铁 | | 废钩 | | 部下 | | 部胆 | |  |
| 乌龟 | 乌拔 | | 机灵 | | 机晾 | | 能源 | | 能贺 | | 手心 | | 手欧 | |  |
| 电扇 | 电格 | | 倒霉 | | 倒敛 | | 鞭子 | | 鞭或 | | 课堂 | | 课把 | |  |
| 半路 | 半哼 | | 焦躁 | | 焦骇 | | 故意 | | 故泄 | | 彩票 | | 彩几 | |  |
| 捣乱 | 捣拴 | | 旗号 | | 旗买 | | 面貌 | | 面涌 | | 石壁 | | 石雪 | |  |
| 必要 | 必宴 | | 号令 | | 号傅 | | 浪费 | | 浪三 | | 山沟 | | 山斥 | |  |
| 被迫 | 被谓 | | 脑海 | | 脑北 | | 抱歉 | | 抱素 | | 情景 | | 情燥 | |  |
| 恭维 | 恭泵 | | 财产 | | 财素 | | 图腾 | | 图赤 | | 从政 | | 从偷 | |  |
| 药店 | 药必 | | 马戏 | | 马收 | | 天际 | | 天吃 | | 面粉 | | 面遁 | |  |
| 拍手 | 拍拱 | | 酒吧 | | 酒偏 | | 扶手 | | 扶米 | | 微笑 | | 微蚯 | |  |
| 报警 | 报谆 | |  | |  | |  | |  | |  | |  | |  |
| **Pseudo P-** | | | | | | | | | | | | | | | |
| prime | | target | | prime | | target | | prime | | target | | prime | | target | |
| 哀思 | | 往怪 | | 共性 | | 律仔 | | 朋友 | | 热娱 | | 相貌 | | 信整 | |
| 霸主 | | 云恍 | | 故土 | | 季厉 | | 偏僻 | | 课跃 | | 香炉 | | 旅诺 | |
| 白蚁 | | 公赌 | | 挂历 | | 学喊 | | 贫寒 | | 住仗 | | 消暑 | | 解远 | |
| 备用 | | 商沥 | | 光景 | | 阴逐 | | 平地 | | 摘彭 | | 小吃 | | 花秦 | |
| 被单 | | 局亚 | | 光芒 | | 游安 | | 评选 | | 弹披 | | 许可 | | 场约 | |
| 表妹 | | 指缩 | | 果脯 | | 灯返 | | 扑面 | | 地毕 | | 学徒 | | 容偶 | |
| 猜谜 | | 难阱 | | 孩童 | | 资谁 | | 朴素 | | 国笼 | | 椅子 | | 呼笋 | |
| 采矿 | | 本林 | | 欢笑 | | 院阮 | | 期望 | | 讲讵 | | 意念 | | 界洒 | |
| 测定 | | 远甘 | | 慌忙 | | 比补 | | 齐集 | | 安讼 | | 阴冷 | | 工君 | |
| 场次 | | 登庵 | | 回答 | | 关韦 | | 欠债 | | 贫咨 | | 用处 | | 冬贩 | |
| 撤离 | | 讲菲 | | 悔改 | | 戒盘 | | 钦佩 | | 聚奠 | | 油库 | | 淡舫 | |
| 成败 | | 产爪 | | 火光 | | 温逗 | | 勤快 | | 付烹 | | 诱发 | | 影淼 | |
| 乘车 | | 市蛮 | | 机舱 | | 秋爸 | | 区分 | | 宝婿 | | 遇难 | | 宝战 | |
| 充足 | | 扫桉 | | 机票 | | 油乱 | | 全长 | | 来凉 | | 原文 | | 比混 | |
| 冲淡 | | 周渚 | | 记得 | | 西姚 | | 全貌 | | 脸涕 | | 战乱 | | 可河 | |
| 出去 | | 结腔 | | 间断 | | 明枉 | | 扰乱 | | 冰巧 | | 支架 | | 楼颂 | |
| 船长 | | 美刚 | | 江水 | | 戏锐 | | 认错 | | 风芭 | | 重活 | | 伤陋 | |
| 纯粹 | | 悲访 | | 交界 | | 生倩 | | 容许 | | 时栽 | | 专长 | | 局同 | |
| 词汇 | | 备匠 | | 胶片 | | 状贫 | | 山寨 | | 奖刚 | | 砖瓦 | | 固朔 | |
| 辞职 | | 法尼 | | 揭开 | | 店呐 | | 烧饼 | | 传昔 | | 庄稼 | | 北棚 | |
| 次数 | | 伴狗 | | 结束 | | 价夸 | | 摄取 | | 本舌 | | 追忆 | | 景多 | |
| 粗壮 | | 旅盂 | | 解除 | | 深姹 | | 师徒 | | 木抽 | | 字母 | | 纠文 | |
| 达标 | | 畅涝 | | 解析 | | 脚顿 | | 实情 | | 名环 | | 走动 | | 植泛 | |
| 答谢 | | 春沸 | | 今朝 | | 保伴 | | 试管 | | 遍兜 | | 坐庄 | | 正难 | |
| 打破 | | 钢浒 | | 救护 | | 泪枝 | | 试想 | | 步特 | | 弯路 | | 生稍 | |
| 大风 | | 氧袒 | | 剧烈 | | 牧汹 | | 手臂 | | 家姗 | | 微观 | | 激巩 | |
| 大爷 | | 沙染 | | 抗拒 | | 矿秧 | | 售出 | | 特周 | | 违纪 | | 晚份 | |
| 当初 | | 蓝萸 | | 渴求 | | 石俭 | | 探测 | | 期番 | | 尾巴 | | 火顷 | |
| 底座 | | 报脑 | | 空手 | | 毒顿 | | 疼爱 | | 背拥 | | 卧室 | | 水维 | |
| 地名 | | 烈酚 | | 理念 | | 黄伤 | | 提拔 | | 测近 | | 磨难 | | 阳缓 | |
| 丢人 | | 光犄 | | 历来 | | 文领 | | 提起 | | 效悚 | | 难处 | | 师腾 | |
| 独唱 | | 卫俯 | | 连夜 | | 首欢 | | 提取 | | 报干 | | 泥沙 | | 花微 | |
| 短文 | | 书准 | | 粮草 | | 敬唐 | | 天职 | | 眼害 | | 排队 | | 金琨 | |
| 对联 | | 地桴 | | 列入 | | 情米 | | 厅堂 | | 路晚 | | 陪伴 | | 轮蛙 | |
| 对手 | | 体肘 | | 裂纹 | | 夜桑 | | 同感 | | 排岗 | | 肺部 | | 城雁 | |
| 反手 | | 首英 | | 麦田 | | 天陛 | | 痛恨 | | 质苏 | | 愤慨 | | 气帼 | |
| 方程 | | 防纠 | | 面食 | | 工冷 | | 头皮 | | 皇挤 | | 风速 | | 银谣 | |
| 放养 | | 原叼 | | 明媚 | | 台金 | | 头球 | | 查难 | | 高兴 | | 整俞 | |
| 肥肉 | | 保玩 | | 明晚 | | 秋涡 | | 涂改 | | 评竹 | | 共鸣 | | 烦记 | |
| 肥沃 | | 山残 | | 模式 | | 黑算 | | 退换 | | 具销 | |  | |  | |
